# Using Tissue Doppler and Speckle Tracking Echocardiography to Assess if Ivabradine Improves Right Ventricular Function

**DOI:** 10.7759/cureus.12920

**Published:** 2021-01-26

**Authors:** Murat Gul, Sinan Inci, Gokhan Aksan, Serhat Sigirci, Pinar Keskin

**Affiliations:** 1 Cardiology, Aksaray University, Faculty of Medicine, Aksaray, TUR; 2 Cardiology, Aksaray University School of Medicine, Aksaray, TUR; 3 Cardiology, Samsun Training and Research Hospital, Samsun, TUR; 4 Cardiology, Sisli Hamidiye Etfal Training and Research Hospital, Istanbul, TUR; 5 Cardiology, Aksaray Training and Research Hospital, Aksaray, TUR

**Keywords:** ivabradine, right ventricular functions, speckle tracking echocardiography

## Abstract

Objective

To evaluate the mid-term effects of ivabradine on right ventricular functions in patients with heart failure.

Methods

A prospective study was conducted on 52 patients who had heart failure in normal sinus rhythm (59% male, age: 64.76 ±12.49 years). Right ventricular functions were measured at baseline, after one month and one year by conventional and tissue Doppler echocardiography imaging. The parameters, right ventricular (RV) longitudinal strain (LS), RV systolic longitudinal strain rate (LSRs), RV early diastolic longitudinal strain rate, and late diastolic longitudinal strain rate, were evaluated by apical four-chamber grayscale imaging through the free wall of RV in accordance with the automated function imaging protocol.

Results

During the follow-up, the pulmonary artery systolic pressure (PASP), RV fractional area change (RVFAC), tricuspid annular plane systolic excursion (TAPSE), myocardial performance index (MPI), E peak, and A peak values were similar to the basal values. While comparing the basal values of the global longitudinal systolic strain (GLS), LS, LSRs, longitudinal strain rate diastolic early filling (LSRe), and longitudinal strain rate diastolic late filling (LSRa), there were no differences in the first month but a significant increase was observed on one-year follow-up (p<0.001).

Conclusion

At the one-year follow-up, the heart failure patients who were given ivabradine treatment showed an improvement in the right ventricular function assessed by the new echocardiographic techniques.

## Introduction

Heart failure is a prevalent health problem and is closely associated with high morbidity and mortality. Despite the advancements in treatment strategies, morbidity and mortality rates remain high [1-2]. The presence of right ventricular dysfunction plays a prominent role in worsening clinical symptoms, prognosis, and functional status in patients with heart failure [3]. The most frequent cause of right heart dysfunction is left-sided heart failure [3].

Ivabradine is a novel selective inhibitor of cardiac If (pacemaker) current, which not only reduces the heart rate but also has anti-ischemic and anti-anginal effects [4]. It has been shown to reduce cardiovascular mortality and hospitalization frequency and improves the quality of life and left ventricular function [5]. Furthermore, ivabradine has improved endothelial function and decreased fibrosis in experimental studies [6-9].

Conventional myocardial assessment by tissue Doppler imaging (TDI) echocardiography has some disadvantages like angle dependence, limited spatial resolution, and one-dimensional deformation analysis. Recently, these limitations have been resolved through advances in two-dimensional speckle tracking echocardiography (STE), and STE has become the preferred technique for this purpose [10].

Studies demonstrating the positive effect of ivabradine on left ventricular remodeling in heart failure patients were conducted [11]. However, currently, there is limited data regarding the effect of ivabradine on right ventricular function. We, therefore, aimed to demonstrate the early and mid-term effects of ivabradine on right ventricular function with conventional and STE.

This work was previously presented as an abstract at the 2nd Cardiovascular Academy Society Congress on October 06, 2016.

## Materials and methods

Patients

This study was conducted prospectively between January 2016 and June 2016 in a single tertiary healthcare center. We studied consecutive 52 adult patients with stable symptomatic chronic heart failure (New York Heart Association (NYHA) class II-III) with left ventricular ejection fraction (LVEF) ≤35 %, and who were in sinus rhythm with a resting heart rate of ≥70 beats/ per minute (bpm). All patients were also receiving guideline-recommended therapies such as beta-blockers (proven or maximum tolerable dose), angiotensin-converting enzyme (ACE) inhibitors (or angiotensin receptor blocker (ARB)), and/or anti-aldosterone agents. The exclusion criteria were as follows: recent (<2 months) myocardial infarction, ventricular or atrioventricular pacemakers, atrial fibrillation or flutter, chronic kidney disease (glomerular filtration rate (GFR) <30), morbid obesity, significant valve disease, ascending aortic aneurism, chronic obstructive pulmonary disease, chronic pulmonary thromboembolism, and poor echocardiographic image quality. The study complies with the Declaration of Helsinki. The research protocol was approved by the institutional review board and informed and signed consent was obtained from all patients.

In accordance with the current treatment algorithm, the starting dose was 5 mg ivabradine twice daily. Afterward, it was up-titrated over 14 days to a target dose of 7.5 mg twice daily unless the heart rate was <60 bpm or symptomatic bradycardia. The dose was adjusted to 7.5, 5, or 2.5 mg twice daily according to the resting heart rate and tolerability. The clinical, electrocardiographic, and echocardiographic measurements of the patients were recorded at baseline and the first and 12th months after the initiation of ivabradine.

Transthoracic echocardiography

Echocardiographic examinations were performed by EPIQ 7 digital ultrasound scanner (Philips Medical System, Eindhoven, Netherlands) in the left lateral decubitus position from multiple windows. All measurements were taken by two experienced cardiologists who were blinded to the clinical status of the patients. The two-dimensional (2D) and pulse wave Doppler echocardiographic studies were performed according to American Echocardiography Association's criteria [12]. A single-derivation electrocardiogram was simultaneously recorded during the examination. The tricuspid annular plane systolic excursion (TAPSE) was determined as the M-mode measurement of tricuspid annulus displacement during systole and diastole [13]. The right ventricular (RV) end-diastolic and end-systolic areas were measured from an apical four-chamber view to calculate the RV fractional area changing (RVFAC) [14]. RV ejection time (RVET) was measured from the interval between the onset of RV ejection to the point of RV outflow cessation. Isovolumetric relaxation time (IRT) was defined as the time interval from the cessation of RV outflow to the tricuspid valve opening. Isovolumetric contraction time (ICT) was defined as the time interval from the tricuspid valve closing to the onset of RV outflow [15]. Myocardial performance index (MPI), a numerical value that can be used to assess ventricular function, was calculated by the formula (ICT + IRT) / RVET [16]. Tissue Doppler measurements were performed in apical four-chamber views, with the sample volume placed at the level of the lateral tricuspid annulus. The systolic velocity (S), early diastolic velocity (E), and late diastolic velocity (A) were obtained. Myocardial acceleration during isovolumic contraction (IVA, m/sec2) and isovolumic contraction myocardial velocity (IVV, cm/sec) were also measured. Left ventricle ejection fraction (LVEF) was calculated with the Teichholz method.

Right ventricular strain and strain rate measurements

**Figure 1:**
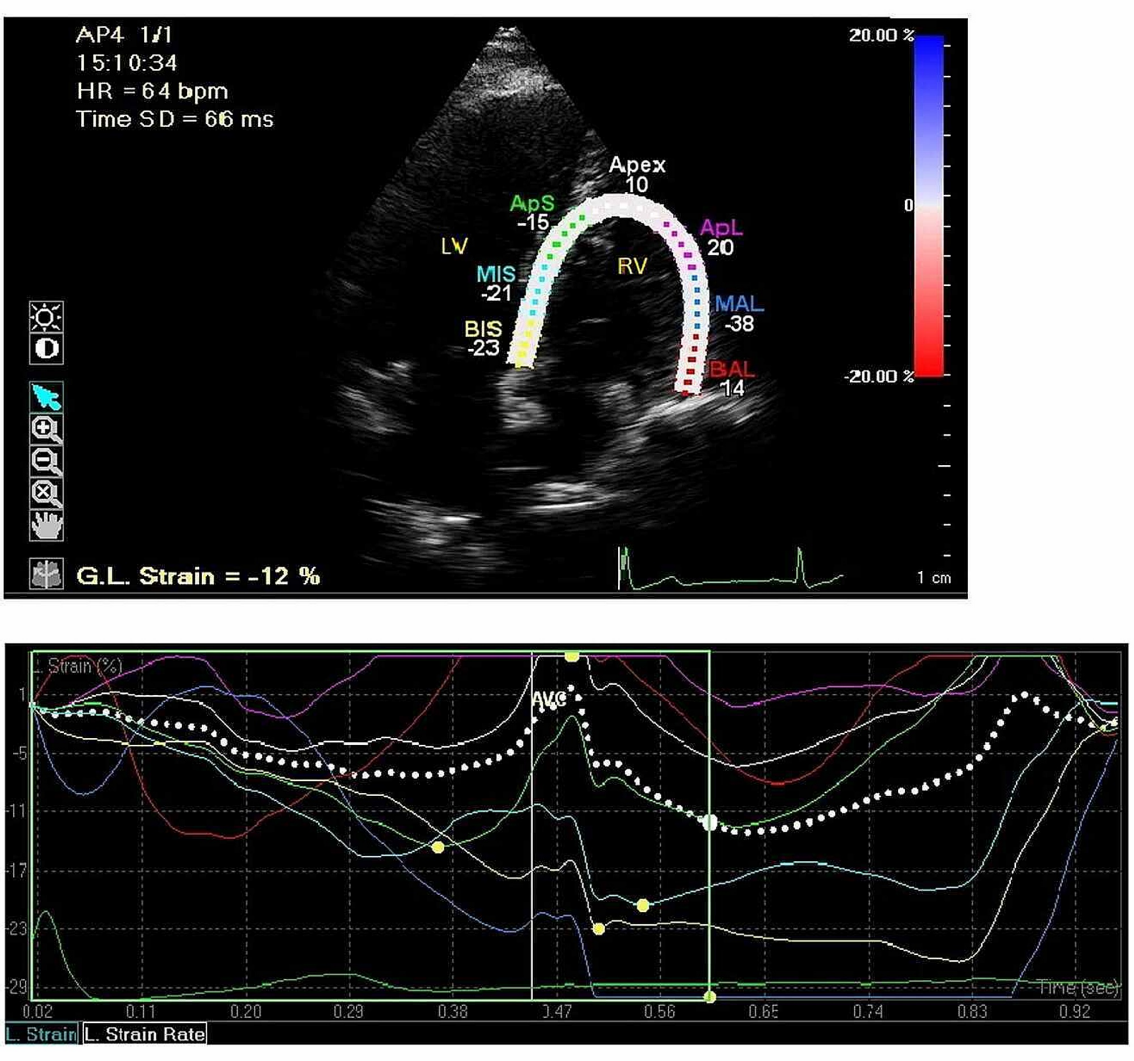
Longitudinal strain (LS) in an apical four-chamber view. Right side average segmental strain displayed in graphs.

For speckle tracking analysis of RV, apical four-chamber view images were obtained using two-dimensional gray-scale echocardiography. The mean frame rate was set as 60 per second (between 50 and 80 per second). Recordings were performed for the images that were having the best endocardial border delineation. Three consecutive cardiac cycles were recorded for all measurements. Afterward, records were processed by tissue Doppler imaging (TDI) data and analyzed offline using dedicated software (QLAB, Philips) (Figure 1). The first step in strain analysis is the determination of the pulmonary valve closing time (PVC) with conventional or automatic function imaging (AFI) methods. We measured the PVC at the end of the T wave with the help of automatic software. Measurements were done by manually placing six to seven endocardial points starting from the basal septum. Using these points, the 2D speckle tracking algorithm followed the endocardium as the region of interest (ROI) to determine the wall thickness along the heart cycle. Three concentric lines showing the endocardial, epicardial, and mid-myocardial layers constitute the ROI. Necessary adjustments were made to measure myocardial thickness by excluding the pericardium. The RV was analyzed with STE by dividing the free wall into three regions, viz., basal, mid, and apical. Also, the strain parameters of each segment were analyzed with the AFI protocol. The global longitudinal systolic strain (GLS), longitudinal strain (LS), longitudinal systolic strain rate (LSRs), longitudinal strain rate diastolic early filling (LSRe), and longitudinal strain rate diastolic late filling (LSRa) parameters were assessed as mentioned earlier [17-18].

Statistical analysis

The results were analyzed with the Statistical Package for the Social Sciences (SPSS) for Windows 15.0 (SPSS Inc., Chicago). Differences in proportions between groups were compared by using the chi-square or Fisher’s exact test, where appropriate. For the normality of the distribution for parameters and for differences for paired variables, the Shapiro-Wilk and Kolmogorov-Smirnov tests were used. The differences among the three measurements such as baseline to one year were evaluated by Friedman's two-way analysis of variance by ranks. When the p-value from the Friedman test statistics is statistically significant, the multiple comparison test was used to know which time points differ from which others. The reliability of the study was assessed for randomly selected 10 patients by using the intraclass correlation coefficient (ICC). Descriptive statistics are summarized as counts and percentages for categorical variables and mean ± standard deviation for continuous variables. Any p-value less than 0.05 was considered statistically significant.

## Results

The baseline demographic and clinical characteristics of the study patients are summarized in Table 1. A total of 52 patients were included in this study. The average age of the patients was 64.76 ±12.49 years, and 59% of them were male. The mean duration of heart failure was 51.05 ±27.98 weeks. The mean heart rate of the patients was 84.15 ±9.61 beats/min while their mean ejection fraction was 27.4 ±6.0. There were 23 patients in NYHA functional class II and 29 in class III (Table 1).

**Table 1:** Clinical characteristics of the study patients NYHA: New York Heart Association

Age (years)	64.76 ±12.49
Gender (female)	21 (41%)
Gender (male)	31(59%)
Body mass index (kg/m^2^)	29.31 ±3.46
Duration of heart failure (weeks)	51.05 ±27.98
NYHA Class 2	23 (44%)
NYHA Class 3	29 (56%)
Hypertension	30 (58%)
Diabetes	24 (46%)
Dyslipidemia	37 (71%)
Cigarette smoking	21 (40%)
Previous stroke	8 (15%)
Peripheral arterial disease	11 (21%)
Heart rate (bpm)	84.15±9.61
Systolic blood pressure (mmHg)	125.38±20.43
Diastolic blood pressure (mmHg)	80.67 ±17.15

The medication details of the patients are demonstrated in Table 2. There were no differences in the medical treatments during the follow-up.

**Table 2:** Comparison of medication parameters at baseline, one-month, and one-year follow-up

	Baseline	One-month	One-year
Beta-blocker	28 (53 %)	28 (53 %)	29 (55 %)
ACE inhibitor or /with Angiotensin receptor blocker	50 (96 %)	50 (96 %)	48 (92 %)
Non-anti-aldosterone diuretics	40 (76 %)	39 (74 %)	37 (70 %)
Anti-aldosterone agents	42 (80 %)	42 (80 %)	42 (80%)
Antiplatelet/anticoagulant	48 (92%)	48 (92%)	46 (88%)
Statins	36 (69%)	36 (69%)	37 (70%)
Cardiac glycosides	10 (20%)	10 (20%)	11 (22%)

A comparison of basal and follow-up (one month and one year) values measured by transthoracic echocardiography is presented in Table 3. There was no significant difference in left ventricular end-diastolic volume (LVEDV) and left ventricular end-systolic volume (LVESV) values at the one-month follow-up, however, a significant decrease at one-year follow-up was observed (p<0.001). There was no difference in LVEF value between the basal value and at the one-month follow-up; however, there was a significant increase at the one-year follow-up (p<0.001). There were no differences in pulmonary artery systolic pressure (PASP), RV fractional area change (RVFAC), tricuspid annular plane systolic excursion (TAPSE), myocardial performance index (MPI), E peak, and A peak values at both the follow-up visits. Deceleration time (DT) was significantly decreased at one-year follow-up.

**Table 3:** Comparison of the baseline, one-month, and one-year follow-up values measured by transthoracic echocardiography LVEDV, left ventricular end-diastolic volume; LVESV, left ventricular end-systolic volume; LVEF, left ventricular ejection fraction; PASP, pulmonary artery systolic pressure; RA, Right atrium; RV, right ventricle; RVFAC, RV fractional area change; TAPSE, tricuspid annular plane systolic excursion P<0.001 comparison of the baseline, one-month, and one-year follow-up values

	Baseline	One-month	One-year
LVEDV, mL	184 ±36.1	182 ±44.6	161 ±47.6
LVESV, mL	122 ±44.2	120 ±51.9	97 ±38.1
LVEF (%) Teicholz	27.4 ±6.0	27.9 ±5.5	30.9 ±5.8
PASP, mmHg	46.23 ±16.93	45.84 ±15.24	45.75 ±14.69
RVFAC, %	36.38 ±4.54	36.57 ±4.36	37.19 ±4.91
TAPSE, mm	15.38 ±2.35	15.65 ±2.69	15.80 ±3.03
Myocardial performance index	0.48 ±0.03	0.49 ±0.05	0.50 ±0.06
E peak, cm/s	43.34 ±5.81	43.50 ±6.08	43.30 ±5.92
A peak, cm/s	45.88 ±4.21	45.53 ±4.72	45.66 ±4.41
Deceleration time, ms	246.05 ±18.70	245.17 ±18.13	241.84 ±20.07

In the TDI parameters measured at the lateral tricuspid annulus, there was a significant increase in systolic Sm and diastolic Em and a decrease in diastolic Am at the one-year follow-up. There was no change in the peak myocardial velocity during isovolumic contraction (IVV) and myocardial acceleration during isovolumic contraction (IVA) parameters during the follow-up (Table 4).

**Table 4:** Comparison of the baseline, one-month, and one-year follow-up pulse tissue Doppler imaging variables measured by transthoracic echocardiography IVV: Peak myocardial velocity during isovolumic contraction; IVA: Myocardial acceleration during isovolumic contraction P<0.001 comparison of the baseline, one-month, and one-year follow-up values

	Baseline	One-month	One-year
Systolic velocity, cm/s	12.34± 1.80	12.57 ±1.25	13.30 ±1.50
Early diastolic velocity, cm/s	10.17 ±1.54	10.53 ±1.69	11.44 ±1.83
Late diastolic velocity, cm/s	12.98 ±1.54	12.90 ±1.49	11.05 ±1.33
Right ventricular IVV (cm/sec)	0.11 ±0.02	0.11 ±0.02	0.12 ±0.02
Right ventricular IVA (m/sec^2^)	2.23 ±0.26	2.24 ±0.25	2.23 ±0.26

Two patients were excluded from the study because of poor image quality. To obtain the average strain and strain rate, the number of non-readable RV segments was within the acceptable limits. A comparison was made for the basal and follow-up values in terms of LSS, LSRs, LSRe, and LSRa values, measured from the basal, mid, and apical aspects of the free wall of the RV. In comparison to the basal parameters, there was no change at the one-month follow-up; however, there was an increase at the one-year follow-up (p<0.001) (Table 5). The two-way random model (Absolute Agreement) of ICC was used to determine the reliability between the measurements taken by two experienced cardiologists. For this purpose, agreement was evaluated for 10 randomly selected patients and the results were found as 0.949 (95% C.I.; 0.897-0.983) and 0.944 (95% C.I.; 0.899-0.974) for LSL and LSR, respectively.

**Table 5:** Comparison of the baseline, one-month, and one-year follow-up longitudinal and global strain-strain rate parameters GLS: Global longitudinal systolic strain; LS: Longitudinal systolic strain; LSRS: Longitudinal systolic strain rate; LSRE: Longitudinal strain rate diastolic early filling; LSRA: Longitudinal strain rate diastolic late filling P<0.001 comparison of the baseline, one-month, and one-year follow-up values

	Baseline	One-month	One-year
GLS (%)	17.48 ±2.06	17.63 ±2.16	18.84 ±1.97
LS %			
Basal (%)	16.44 ±2.03	16.57 ±2.20	17.78 ±1.81
Mid (%)	17.26 ±2.07	17.35 ±2.20	18.54 ±1.97
Apical (%)	18.19 ±2.06	18.25 ±2.34	19.60 ±1.99
LSRS			
Basal, 1/s	1.66 ±0.11	1.66 ±0.11	1.71 ±0.11
Mid, 1/s	1.63 ±0.11	1.63 ±0.11	1.68 ±0.10
Apical, 1/s	1.55 ±0.10	1.55 ±0.10	1.60 ±0.11
LSRE			
Basal, 1/s	1.87 ±0.12	1.87 ±0.12	1.90 ±0.13
Mid, 1/s	1.89 ±0.12	1.89 ±0.12	1.92 ±0.13
Apical, 1/s	1.75 ±0.14	1.73 ±0.15	1.78 ±0.16
LSRA			
Basal, 1/s	1.48 ±0.11	1.47 ±0.11	1.50 ±0.12
Mid, 1/s	1.47 ±0.13	1.48 ±0.12	1.55 ±0.13
Apical, 1/s	1.38 ±0.12	1.39 ±0.11	1.42 ±0.12

## Discussion

In this study, ivabradine showed functional improvement in the right ventricle but not a morphological one after one year of treatment as demonstrated by the new echocardiographic techniques. There are several methods for routine practice to evaluate the right ventricular function. Two-dimensional strain, which is done by using standard two-dimensional views, can perform strain measurements with speckle tracking. Moreover, in comparison to conventional strain measurements, it has low angle dependency and high reproducibility. STE can measure intramyocardial velocities in three dimensions (longitudinal, radial, and circumferential) and shows very low inter- and intraobserver variability. Recent studies have demonstrated that this method is practical and efficient for the evaluation of the right ventricular function in routine practice [19-20]. Simsek et al. investigated the right ventricular function in patients with nasal polyposis [20]. RV basal, mid and apical peak systolic strain, and velocities were found to be lower in patients with nasal polyposis. In this study, when we compared the basal, one-month, and one-year values of peak systolic strain and velocity parameters in patients with heart failure who were having ivabradine treatment, we observed no difference in GLS, regional strain (basal-mid-apical), and the LSRS (basal-mid-apical), LSRE (basal-mid-apical), LSRA (basal-mid-apical) values at the one-month follow-up but there was a significant increase at the one-year follow-up.

In recent years, the importance of the hemodynamic stability of the right ventricle (RV) for the preservation of the other organ functions has been realized. The isolated right ventricular failure has higher mortality than the isolated left heart failure [21]. The most common etiology is the development of right heart failure secondary to left heart failure. We aimed to demonstrate a favorable effect of ivabradine on right ventricular function, which has already known to have a favorable effect on left ventricular function. Increased pulmonary capillary wedge pressure in left heart failure by increasing pulmonary artery pressure also increases right ventricular afterload, which, in turn, impairs right ventricular function. The right ventricle is vulnerable to increased afterload due to its low wall mass and high wall stress [22-23].

Ivabradine is a new pharmacological agent, which is a specific inhibitor of the If channel on the sinoatrial node [24]. The only known effect of this agent is decreased heart rate. However, details of the favorable contribution of ivabradine in heart failure remain unknown. Ivabradine treatment has been shown to improve cardiac remodeling [25-26]. In our study, at the end of one-year of ivabradine treatment, LVEDV, LVESV, and EF were increased significantly. Although the main mechanism of ivabradine is to decrease the heart rate, it might affect remodeling by different mechanisms. However, some clues can be obtained through experimental studies. In rat models with chronic heart failure, ivabradine increases cardiac output and LV function [6]. These changes are related to the optimization of energy distribution at the myocyte function, extracellular matrix, and tissue level. In another study, ivabradine was shown to contribute to the remodeling by structural and electrophysiologic conversion regulation [27]. A similar effect of ivabradine was also shown in rat models with advanced heart failure [7-9]. In these studies, the possible mechanisms were supposed to be, in addition to an improvement in endothelial function, regulation of the sympathetic nervous system, activation of the local renin-angiotensin-aldosterone system (RAAS), and decrease in fibrosis. In canine models, ivabradine displayed a positive effect in sarcoplasmic reticulum calcium turnover [28]. The results of all these experimental studies have demonstrated the favorable effects of ivabradine on cardiac remodeling. In our study, ivabradine functionally improved the right ventricle but not morphologically. The underlying mechanism can be related to the improvement of the left ventricular function that decreases the right ventricular afterload. However, this mechanism was rejected because the improvement was at the tissue level but not in the morphology as shown by experimental studies. Ivabradine, by controlling tissue energy coupling, regulating sarcoplasmic reticulum Ca turnover, decreasing fibrosis, and regulating hormonal turnover, improves right ventricular remodeling. This may have some effect on decreasing morbidity and mortality in patients with heart failure.

Limitations

This study should be evaluated in light of some limitations. First, our study was a non-randomized, two-centered study composed of a limited number of patients. Additional clinical randomized trials with greater participation can offer more reliable statistical data. Second, the measurements used to predict RV dysfunction were not independent parameters. Furthermore, RV functions were measured by only longitudinal strain and strain rate parameters but not at the radial and circumferential levels. Although radial and circumferential measurements have been extensively studied in LV strain analysis, their importance in the right ventricle is not well-known. Also, their estimation is more technically challenging than the LV due to the relatively thinner wall and complex geometry of the RV. Finally, the AFI protocol is software primarily produced for LV strain analysis. However, we used the AFI protocol for RV strain analysis depending on an experimental study wherein its utility was shown in RV strain analysis [29]. Nevertheless, these limitations might have been minimized since this was a follow-up study.

## Conclusions

In the current study, a one-year follow-up of patients with heart failure receiving ivabradine treatment was investigated by conventional echocardiography and STE. The right ventricle showed functional improvement but did not change morphologically. We believe that these findings should be tested with prospective and larger studies.
